# Accuracy of large language models in head and neck cancers: a comparative analysis of ChatGPT and Gemini in TNM staging and clinical decision support

**DOI:** 10.3389/fonc.2026.1828538

**Published:** 2026-05-08

**Authors:** Deniz Baklaci, Hüseyin Işık, Duygu Erdem, Gökhan F. Kılıç

**Affiliations:** Faculty of Medicine, Zonguldak Bulent Ecevit University, Zonguldak, Türkiye

**Keywords:** ChatGPT, clinical decision support systems, gemini, head and neck cancers, TNM staging

## Abstract

**Objective:**

Large Language Models (LLMs) are increasingly integrated into oncological workflows. This study evaluated and compared the performance of ChatGPT-4o and Gemini 1.5 Pro in TNM staging and treatment planning for head and neck malignancies across diverse anatomical sites.

**Materials and Methods:**

A retrospective analysis was performed on 180 patients with head and neck cancer. Clinical data were processed through structured prompts to simulate real-world clinical inquiries. AI-generated TNM stages and treatment protocols were compared against AJCC 8th Edition guidelines and expert multidisciplinary tumor board decisions. The analysis focused on diagnostic accuracy, inter-rater agreement, and the impact of anatomical complexity on model performance.

**Results:**

Both models demonstrated comparable proficiency in TNM staging accuracy (ChatGPT: 75.6%, Gemini: 75.0%), showing substantial agreement with expert standards (x^2^ = 0.000, p = 1.000). However, a significant divergence was observed in treatment planning; Gemini achieved a 78.9% accuracy rate, significantly outperforming ChatGPT’s 71.7% (p=0.043 (x^2^ = 4.114). Notably, ChatGPT’s staging performance was sensitive to tumor localization, with decreased precision in anatomically complex regions such as the oropharynx and paranasal sinuses (p = 0.034, Cramer’s V = 0.291). Conversely, Gemini demonstrated more robust spatial reasoning across different subsites.

**Conclusion:**

While both LLMs provide reliable staging support, Gemini exhibits superior clinical reasoning in synthesizing multidimensional data into actionable treatment recommendations. However, a staging error rate of 25% remains a critical concern, potentially leading to inappropriate clinical pathways. These models should be viewed as auxiliary tools within an ‘augmented intelligence’ ecosystem, integrated with imaging and multidisciplinary inputs, rather than independent decision-makers. Strict expert supervision is mandatory to prevent subsite-specific errors from impacting surgical and oncological outcomes.

## Introduction

Head and neck cancers (HNC) are the generic term used to describe a heterogeneous group of malignancies, with high death and morbidity rates worldwide (1). The  maximum of therapeutic success in these tumors is based on the accuracy of diagnosis and tailoring treatment planning accordingly (2). In the oncological setting, the qualification of the AJCC 8th edition has marked a change of paradigm; inclusion criteria with p16 expression and depth invasion in staging systems have improved predictive accuracy but also increased complications in the cognitive process behind clinical decision (3, 4).

This growing cognitive load in the management of patients has led to Artificial Intelligence (AI), and its subfield Large Language Models (LLMs) in particular, becoming a central theme of discussion within the medical community (5, 6). Generative AI models are now being deployed within clinical practice for oncology, and offer the potential for condensing multifaceted medical data and robust clinical support systems (7, 8). Early studies in the literature suggest that LLMs may have value in reviewing radiologic reports, for TNM staging and devising treatment plans (9–11). But all of them critically need to be thoroughly studied in such high stakes areas as oncology, where there is  no tolerance for errors, and compliance with clinical practice patterns among these models, as well as the performance gaps between different models, still need intensive exploration (1, 12).

Recent comparative studies suggest that leading models such as ChatGPT and Gemini achieve variable success rates between  different oncological subdisciplines. For example, investigation in gastrointestinal cancer and management revealed that the model’s performance varied among different cases for adherence to NCCN guidelines (12, 13); while in neuro-oncology analyses have shown promising results for accuracy, completeness of records, and clinical usefulness (14, 15). In the more narrow area of interest of head and neck surgery, systematic reviews have shown that on responses to ChatGPT-4 oral cancer questions (i.e., does this look like a tumor) and thyroid nodule risk analysis are reliable but warned clinicians of the hallucinations risks (4, 16, 17).

The ability of models including ChatGPT-4o and Gemini 1.5, to produce research methods, provide clinical decision support in laryngology as well as otolaryngology, demonstrates these tools’ evolutionary path (10, 18, 19). Comparative studies focusing on advanced stage HNC, in particular, have questioned the capability of ChatGPT-4 and Gemini Advanced to understand complex oncological situations other than easy to understand text based analysis (1, 20). Additionally, recent studies mainly performed with up to date models such as DeepSeek and Perplexity in salivary gland cancer (SGC), demonstrate the fast growing digitalization of oncology literature (20, 21). Moreover, the precision of AI-generated response to post treatment quality of life issues raises the possibility that these models could be used as a critically beneficial aide for clinician, but also patient driven care (22, 23).

On the other hand, most available studies are model specific or generalize from small samples. In head and neck oncology, clinical judgment required to integrate radiological data with clinical examination to call AJCC 8th edition staging and subsequent surgical/oncological management is significant. There is a continued need for larger evaluation studies to have ChatGPT and Gemini compared within a wide patient set for TNM staging accuracy and adherence to the guidelines (6, 24). While recent pilot studies, such as the one by Lorenzi et al., have explored LLM performance using a limited number of hypothetical clinical vignettes (e.g., n=5), there remains a critical gap in understanding how these models perform across a large, heterogeneous, and real-world patient population (1). Our study addresses this gap by evaluating 180 actual clinical cases, providing a robust statistical foundation to compare ChatGPT (GPT-4o model) and Gemini (1.5 Pro model) specifically in head and neck oncology.

The primary objective of this study is to evaluate the performance of ChatGPT and Gemini models in staging and clinical decision-making processes in head and neck cancer using real data, according to the AJCC 8 TNM staging system. To this end, 180 clinical cases were analyzed to assess the accuracy of these models and statistically compare their performance based on parameters including tumor location and demographic variables. This study presents one of the largest statistical comparisons to date of the staging and surgical decision support performance of these two advanced artificial intelligence models, which have been widely researched in the literature, in the field of head and neck oncology.

## Materials and methods

The study was designed a retrospective, comparative, cross-sectional diagnostic accuracy type of study. The main purpose was to assess and compare the performance of two major LLMs (OpenAI’s ChatGPT-4o and Google’s Gemini 1.5 Pro) in challenging oncological tasks where TNM staging and treatment planning are involved for head and neck cancers.

**Figure 1:**
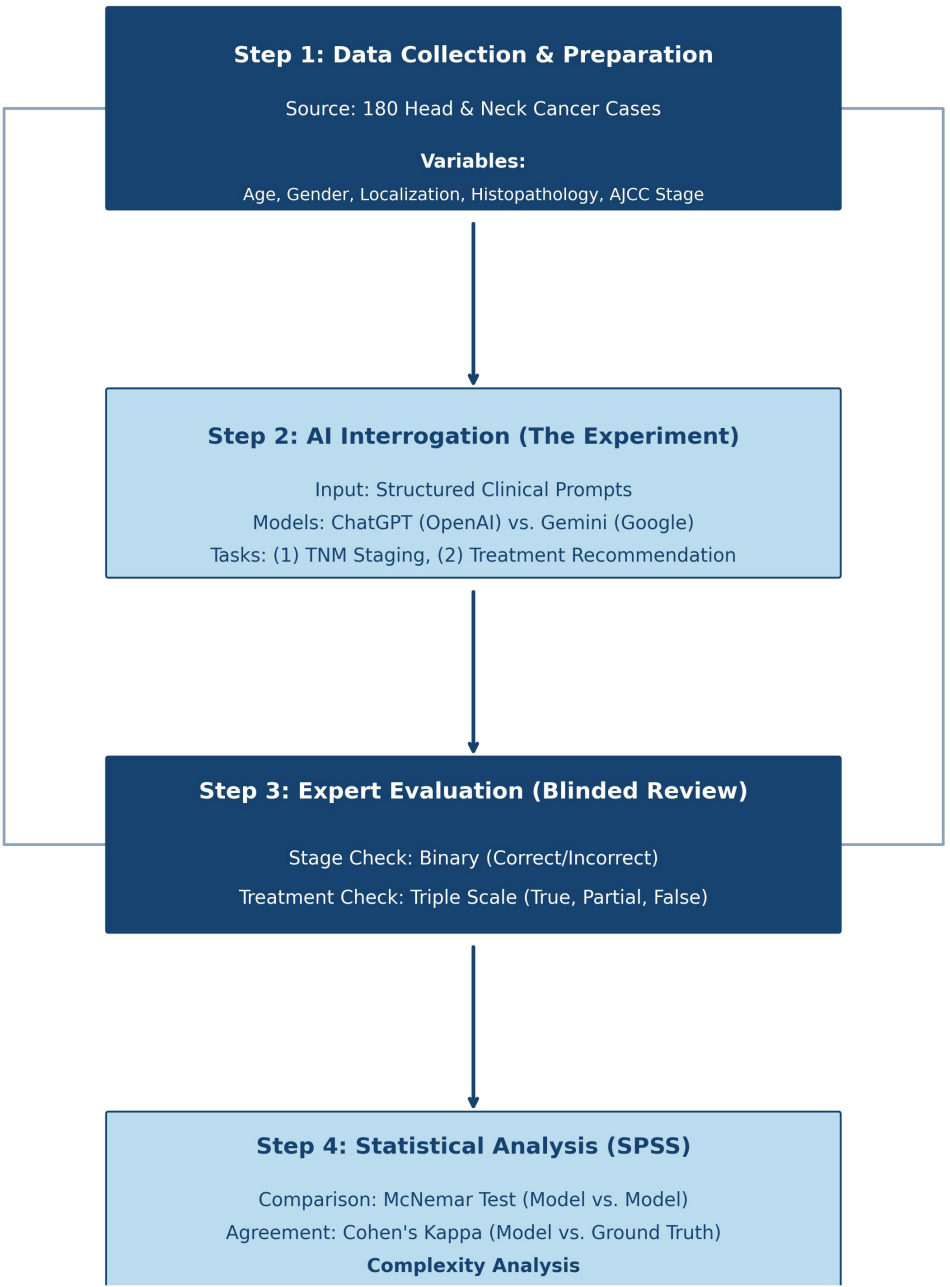
The methodological flowchart.

The protocol of this study was approved by the Ethics Committee of Bülent Ecevit University (Ethics no. [03/09/2025] 2025-15) and was performed in accordance with the principles of the Declaration of Helsinki. Inasmuch as the current investigation employed anonymized retrospective clinical data and did not constitute human participant research or include interventional activity on patient subjects for which any identifiable information derived from those subjects was obtained, it was performed in accordance with the institution’s system of record. The methodological flowchart is given in **Figure 1**.

### Patient selection and eligibility criteria

This retrospective study included consecutive patients who underwent surgery for head and neck malignancies at our clinic between June 2020 and June 2025. All included cases had been discussed in the institutional multidisciplinary head and neck tumor board, which served as the basis for the reference clinical staging and treatment decision process. Patients were excluded if they had incomplete clinical, radiological, pathological, or staging data that precluded standardized case prompt generation, recurrent disease, previous oncologic treatment before admission, unknown primary tumor, or lack of tumor board evaluation. The final cohort comprised 180 eligible cases. As this was a retrospective real-world consecutive-case study, no formal *a priori* power analysis was performed; instead, all eligible patients within the predefined study period were included.

### Patient population and dataset characterization

A total of 180 patients diagnosed with various head and neck malignancies were included in the study. The dataset was curated to represent the clinical reality and heterogeneity of head and neck oncology. The cohort consisted of 129 males (71.7%) and 51 females (28.3%), reflecting the global epidemiological male predominance in these cancers. The mean age was 66.2 ± 13.9 years, with a median age of 69, indicating a predominantly elderly population consistent with the natural incidence of these diseases.

To challenge the AI’s spatial and anatomical reasoning, cases were selected from diverse subsites, including the glottis, supraglottic/transglottic regions, parotid gland, oral cavity, nasopharynx, oropharynx, and paranasal sinuses. The dataset included Squamous Cell Carcinoma as the most frequent type, along with rarer pathologies such as Adenoid Cystic Carcinoma, Mucoepidermoid Carcinoma, and Acinic Cell Carcinoma.

### Clinical standards

The ground truth of each case originated from the consensus obtained by a multidisciplinary tumor board (MDTB) and was confirmed by two experienced senior otorhinolaryngologists who had more than 15 years of experience in head and neck oncology. Tumor was staged according to the  AJCC’s 8th edition. Therapeutic recommendations were compared to the National Comprehensive Cancer Network (NCCN) Guidelines and decisions taken by the tumor board in terms of surgical margins, neck dissection, adjuvant therapy. In instances where a discrepancy occurred between the institutional MDT decision and the NCCN Guidelines, the NCCN recommendations were set as the primary reference for grading AI accuracy. This ensured that the models were evaluated against universal evidence-based standards rather than institution-specific clinical variations.

### Data input and AI interrogation protocol

To ensure methodological relevance, the study utilized the most stable and advanced versions available during the data collection period (September 2025): OpenAI’s ChatGPT-4o and Google’s Gemini 1.5 Pro. The queries were processed using a Structured Clinical Prompting technique. This approach was intentionally selected to simulate the real-world experience of a frontline clinician who requires rapid, guideline-consistent decision support without the need for complex prompt engineering. By providing standardized clinical data, we aimed to evaluate the models’ inherent medical reasoning and their adherence to AJCC/NCCN standards in a typical clinical workflow. Each case was entered into a separate, new chat session to prevent contextual carryover and to maintain the independence of each diagnostic and treatment recommendation. The same standardized prompt structure was utilized for both models throughout the interrogation process. A sample text was generated as follows:

“A 79-year-old male patient presented with a lesion on the right lateral aspect of the lower lip, which was diagnosed as Squamous Cell Carcinoma (SCC). Clinical examination revealed an approximately 1 cm lesion, with no palpable lymphadenopathy detected in the neck. Neck CT showed a soft tissue density area approximately 16 mm in diameter in the right lower region, while cervical lymph nodes were observed with a short axis not exceeding 1 cm. Other ENT structures, including the nasopharynx, larynx, and thyroid, were unremarkable. Thoracic CT was normal. What is the clinical stage of the tumor according to the AJCC 8th edition? What is the surgical treatment approach for this patient?” (**Table 1**).

**Table 1:** Comparison of NCCN guidelines and AI models (chatGPT-4o and gemini 1.5 Pro) in the clinical staging and treatment planning of a patient with lower Lip SCC.

Feature/Source	NCCN/national head and neck tumor council	Chat GPT - 4o (AI Model)	Gemini 1.5 Pro (AI Model)
I. Clinical Staging (AJCC 8th Ed.)	cT1N0M0	Clinical stage (AJCC 8th ed.)For lip (vermilion)/oral cavity SCC, AJCC 8 uses tumor size + depth of invasion (DOI) for the T category. • Clinically the lesion is ~1 cm (CT suggests up to ~16 mm, still < 2 cm). • No palpable nodes and CT shows no clearly pathologic cervical nodes (short axis ≤ 1 cm) → cN0. • Thoracic CT normal → cM0. So, if the DOI is ≤ 5 mm (which is typical for many ~1 cm early lip lesions), the clinical stage is: • cT1 cN0 cM0 → Stage I If DOI later proves to be > 5 mm (up to 10 mm), the tumor would upstage to cT2, which would make it Stage II (T2N0M0). DOI is therefore the key missing piece if you want an unambiguous AJCC 8 T category pre-op.	Based on the clinical details and the summary provided, here is the Clinical Staging and Surgical Treatment Approach for the 79-year-old patient with squamous cell carcinoma (SCC) of the lip. 1. Clinical Staging (AJCC 8th Edition) T-Stage (Primary Tumor): cT1 • The lesion is clinically 1 cm and measures 1.6 cm on CT. Since the greatest dimension is ≤ 2 cm, it is classified as T1. • Note: The AJCC 8th edition incorporates Depth of Invasion (DOI). If the final pathological DOI is found to be > 5 mm, the tumor would be upstaged to pT2. N-Stage (Regional Lymph Nodes): cN0 • There are no palpable lymph nodes, and CT shows cervical lymph nodes are < 1 cm with no suspicious features. M-Stage (Distant Metastasis): cM0 • Thorax CT is normal. Overall Clinical Stage: Stage I
II. Surgical Treatment (Lip, Reconstruction, and Neck Management)	Tumor resection +/- right level 1–2 neck dissection	Surgical treatment approach 1) Primary tumor (lip) • Wide local excision/wedge (V or W) resection of the lower lip lesion with adequate margins (commonly ~1 cm clinically for mucosal SCC when feasible, with intraoperative frozen sections if available/needed). Reconstruction: • If the defect is ≤ ~1/3 of lip width: primary closure is usually appropriate. • If larger: local flap options (e.g., Abbé/Estlander, Karapandzic, etc.) depending on defect size/location. 2) Neck management (clinically N0) • For an early, small lateral lower-lip SCC with cN0, standard management is no therapeutic neck dissection. • Elective neck treatment (e.g., sentinel lymph node biopsy or selective neck dissection levels I–III) is generally considered only if high-risk features are present, especially: • DOI > 4–5 mm (commonly used threshold), • poor differentiation, perineural/lymphovascular invasion, close/positive margins, etc. Practical plan: excision + close neck observation, escalate if needed.	2. Surgical Treatment Approach A. Management of the Primary Tumor (Lip) • Procedure: Wide Local Excision (Wedge Resection). • Margins: At least 5 mm. • Reconstruction: Primary Repair. • Likely <30% lip width → direct closure. • Can be under local anesthesia. B. Management of the Neck (cN0) • Recommendation: Observation (“Wait and Watch”). • Low metastasis risk (<10–15%). • Follow-up with exam + ultrasound. • If DOI >5 mm or high-risk → selective neck dissection. Summary Plan: Wedge Resection + Primary Repair + Observation.

The AI outputs were categorized and graded by the expert panel as follows:

- Staging Accuracy:
- True: Exact match with the AJCC ground truth.
- False: Any deviation in T, N, or M categories.
- Treatment Accuracy:
- True: Full alignment with NCCN/Tumor Board guidelines.
- Partial: Correct primary modality but missing crucial details.
- False: Inappropriate or contraindicated treatment recommendations.

This pragmatic three-tier categorization (true/partial/false) was adopted to reflect commonly used approaches in prior LLM clinical-evaluation studies that assess guideline concordance and clinical usefulness, where outputs may be fully concordant, partially concordant (i.e., correct primary modality but incomplete), or discordant with guideline-based care (1, 12, 13, 24). In this context, ‘partial’ was used for recommendations that identified the correct overall treatment modality but omitted key guideline-dependent elements (e.g., elective neck management, margin strategy, or adjuvant therapy indications.

### Statistical analysis

Statistical analysis was performed using SPSS Statistics v26.0. Given the categorical and non-parametric nature of the data, the following advanced tests were employed:

- Comparative Performance: To determine if there was a significant difference between ChatGPT and Gemini’s binary accuracy (Correct vs. Incorrect), the McNemar test was used for paired nominal data.
- Concordance Analysis: Cohen’s Kappa (k) coefficient was calculated to measure the agreement between the AI models and the AJCC ground truth. The k values were interpreted as: 0.61–0.80 (Substantial) and 0.81–1.00 (Almost Perfect).
- Correlation with Clinical Factors: To explore whether anatomical complexity or tumor type influenced AI performance, Pearson’s Chi-Square (x^2^) test was applied.
- Effect Size: To quantify the strength of the relationship between clinical variables and model failure, Cramer’s V was utilized. A Cramer’s V value of approximately 0.30 was considered a moderate effect size.
- Significance: A two-tailed p value of < 0.05 was considered statistically significant for all tests.

## Results

### Descriptive characteristics of the dataset

The study cohort comprised 180 patients with a significant male predominance (n=129, 71.7%) and a mean age of 66.2 ± 13.9 years. The anatomical distribution was highly heterogeneous, with the most common primary sites being the glottis (17.3%) and the parotid gland (9.5%). Pathological analysis revealed that while SCC was the dominant morphology, the dataset included a robust variety of minor salivary gland tumors and rare malignancies, ensuring a high stress test environment for the AI models. The baseline demographic and clinical characteristics of the study population are given in **Table 2**.

**Table 2:** The baseline demographic and clinical characteristics of the study population.

Characteristic	Value
Age (years)	
Mean ± SD	66.2 ± 13.9
Median	69 (26–92)
Gender, n (%)	
Male	129 (71.7%)
Female	51 (28.3%)
Most Frequent Localizations, n (%)	
Oral cavity	56 (31.1%)
Larynx	50 (27.8%)
Skin	36 (20.0%)
Salivary gland	21 (11.7%)
Oropharynx	9 (5.0%)
Nasopharynx	6 (3.3%)
Paranasal sinus	2 (1.1%)
AJCC Stage, n (%)	
T1N0M0	48 (26.67%)
T2N0M0	41 (22.78%)
T3N0M0	21 (11.67%)
T1aN0M0	11 (6.11%)
T4aN0M0	9 (5.00%)
T2N1M0	7 (3.89%)
T4aN1M0	4 (2.22%)
T1bN0M0	4 (2.22%)
T2N3bM0	3 (1.67%)
T3N1M0	3 (1.67%)
T1N1M0	3 (1.67%)
T2N2bM0	3 (1.67%)
T4aN2bM0	2 (1.11%)
T3N2bM0	2 (1.11%)
T4aN2cM0	2 (1.11%)
T2N2M0	2 (1.11%)
T4aN2bM0	2 (1.11%)
TisN0M0	2 (1.11%)
T4bN0M0	1 (0.56%)
T2N3M0	1 (0.56%)
T1N2M0	1 (0.56%)
T3N2M0	1 (0.56%)
T4N2M0	1 (0.56%)
T1N0M0	1 (0.56%)
T3N2bM0	1 (0.56%)
T3N1N0	1 (0.56%)
T1N2bM0	1 (0.56%)
T1N2cM0	1 (0.56%)
T2N2cM0	1 (0.56%)

### TNM staging performance

Both ChatGPT and Gemini demonstrated a high degree of proficiency in TNM staging, with nearly identical overall accuracy rates:

- ChatGPT Staging Accuracy: 75.6% (n=136/180)
- Gemini Staging Accuracy: 75.0% (n=135/180)

McNemar’s test confirmed that there was no statistically significant difference between the two models in terms of staging precision (x^2^ = 0.000, p = 1.000). Discordant pair analysis showed that ChatGPT was correct while Gemini was incorrect in 21 cases, while the reverse occurred in 20 cases.

Furthermore, Cohen’s Kappa (*k*) analysis indicated a substantial level of agreement with the ground truth for both models. ChatGPT achieved a *k* of 0.706 (p < 0.001), while Gemini followed closely with a *k* of 0.697 (p < 0.001). These findings suggest that for structured classification tasks like TNM staging, both LLMs perform with high reliability. The comparative accuracy and reliability analysis of ChatGPT and Gemini are given in **Table 3**.

**Table 3:** The comparative accuracy and reliability analysis of Chatgpt and Gemini.

Metric	ChatGPT	Gemini	p-value (McNemar)
Staging Accuracy (%)	75.6%	75.0%	1.000
Treatment Accuracy (%)	71.7%	78.9%	0.043*
Kappa (*k*) vs. Ground Truth	0.706	0.697	< 0.001

The asterisk (*) denotes statistical significance, indicating a p-value < 0.05.

### Treatment planning

In contrast to staging, the models’ ability to formulate treatment recommendations showed a statistically significant difference.

- Gemini achieved a “True” treatment recommendation rate of 78.9%, whereas ChatGPT remained at 71.7%.
- Notably, ChatGPT’s “Partial” recommendation rate was significantly higher (28.3%) compared to Gemini (20.6%).

McNemar’s test for paired treatment accuracy yielded a p-value of 0.043 (x^2^ = 4.114), indicating that Gemini significantly outperformed ChatGPT in clinical decision-making. Discordant analysis revealed that in 24 cases where ChatGPT failed to provide a full treatment plan, Gemini was successful, whereas the opposite occurred in only 11 cases.

### Impact of clinical factors

The influence of anatomical and pathological complexity on AI performance was evaluated using Pearson’s Chi-Square and Cramer’s V and given in **Table 4**.

**Table 4:** The correlation between anatomic localization and model performance.

Model performance	Pearson χ2	p-value	Cramer’s V
Localization × ChatGPT Staging Accuracy	15.194	0.034*	0.291
Localization × Gemini Staging Accuracy	10.044	0.186	0.236
Localization × ChatGPT Treatment Accuracy	13.064	0.161	0.269
Localization × Gemini Treatment Accuracy	25.362	0.031*	0.265

The asterisk (*) denotes statistical significance, indicating a p-value < 0.05.

ChatGPT’s staging accuracy was significantly affected by tumor localization (p = 0.034, Cramer’s V = 0.291). Specifically, accuracy dropped in anatomically complex regions like the oropharynx and paranasal sinuses. Conversely, Gemini’s staging accuracy did not show a statistically significant correlation with localization (p = 0.186), suggesting higher robustness across different anatomical sites. Gemini’s treatment recommendations, however, were sensitive to localization (p=0.031, Cramer’s V = 0.265), reflecting the inherent complexity of surgical vs. non-surgical decisions in specific regions like the larynx or oral cavity. Neither model’s performance was significantly impacted by the histological type of the tumor (p > 0.05), indicating that their logic is primarily driven by staging parameters rather than tissue morphology.

## Discussion

The integration of LLMs into clinical oncology represents a paradigm shift in decision support systems, yet their reliability in high stakes fields like head and neck surgery remains a subject of intense academic scrutiny. This study provides a comprehensive comparative analysis of ChatGPT and Gemini in the management of head and neck malignancies, revealing that while both models exhibit substantial agreement with gold standard AJCC staging, Gemini demonstrates a statistically significant superiority in formulating multidiscipinary treatment plans. This finding aligns with the evolving literature suggesting that LLMs are transitioning from simple data retrieval tools to complex reasoning engines (6, 7). However, the variability in performance across different anatomical sites and clinical tasks underscores the necessity for rigorous validation before these tools can be safely integrated into the MDTB workflow. Our findings significantly extend the preliminary results reported in recent literature. Unlike previous studies that relied on a handful of simulated cases (1, 19), the use of 180 real-world cases in this study allowed for a more granular sub-site analysis. This approach revealed that model reliability is highly dependent on anatomical localization, a nuanced finding that smaller-scale studies were unable to demonstrate statistically. By moving beyond small, hypothetical samples, our results provide a more accurate reflection of the clinical complexities encountered in multidisciplinary tumor boards.

The results regarding TNM staging accuracy indicate that for structured, rule based tasks, the models perform at a comparable level. This high level of concordance is supported by Cohen’s Kappa values, reflecting substantial agreement with the AJCC 8th Edition. These findings are consistent with Kayaalp et al., who confirmed the reliability of LLMs in head and neck cancer staging, noting that the models effectively process TNM parameters when provided with structured clinical data (3). Similarly, da Silva highlighted the potential of deep learning pipelines for staging, suggesting that the logic required for AJCC classification is well within the current capabilities of top tier generative AI (9). Nevertheless, the 25% error rate observed in our study suggests that the human in the loop requirement remains indispensable to prevent staging errors that could lead to under or over treatment. This necessity for expert oversight is further emphasized by Yan et al., who reported an 83.3% accuracy rate for ChatGPT-4 in staging recurrent or metastatic HNSCC, while noting critical ‘reasoning errors’ in differentiating complex T4a and T4b lesions (25). Such findings reinforce our observation that even high-performing models can falter in the nuanced anatomical details required for precise TNM classification.

The most striking finding of this study is the significant divergence in treatment planning performance, where Gemini outperformed ChatGPT. This superiority is particularly noteworthy as it contradicts some recent studies in other oncological fields. For instance, Li et al. reported that ChatGPT outperformed Gemini in gastric cancer decision-making (13), whereas Bresler et al. found comparable performance between Gemini and ChatGPT across six gastrointestinal cancers (12). The superior performance of Gemini in our head and neck cohort may be attributed to its more robust integration of NCCN guidelines and its ability to handle the partial or incomplete recommendations that frequently hindered ChatGPT. As noted by Lorenzi et al. in their comparison of ChatGPT and Gemini Advanced for head and neck malignancies, the choice of model can significantly impact clinical utility, with Gemini often showing a more nuanced understanding of advanced disease management (1). The landscape of inter-model performance in oncology remains highly dynamic and often site-specific. Complementing our findings, Camalan et al. (2025) recently demonstrated in a guideline-based study that DeepSeek V3 significantly outperformed ChatGPT o1 in treatment planning accuracy (80% vs. 62%, p=0.04) (26). In contrast, Erim et al. reported that ChatGPT-4 could generate highly reliable decisions comparable to MDTB standards specifically for primary laryngeal cancer, showing no statistically significant difference from expert board decisions in therapeutic management (27). These varying results across different models and cancer sites, including our own observation of Gemini’s clinical edge (p=0.043), underscore that model selection and versioning are decisive factors in capturing the nuances of clinical decision-making.

The analysis of clinical factors revealed that ChatGPT’s staging accuracy was significantly influenced by anatomical localization, with performance dropping markedly in complex regions like the oropharynx and paranasal sinuses. This localization sensitivity suggests that ChatGPT may struggle with the intricate spatial and regional lymphatic drainage patterns characteristic of these sites. This observation is echoed by Schmidl et al., who noted that the anatomical complexity of primary head and neck cases often tests the limits of AI diagnostic accuracy (8). In contrast, Gemini’s staging accuracy remained stable across various sites, indicating a more robust spatial reasoning capacity. However, Gemini’s treatment recommendations were sensitive to localization, likely reflecting the inherent complexity of choosing between organ preservation protocols and radical surgery in diverse subsites like the larynx versus the oral cavity (19). These findings collectively suggest that LLMs should not be conceptualized as universal expert clinicians or general practitioners capable of uniform performance across all scenarios. Instead, these data highlight that their reliability is highly dependent on the anatomical context. Therefore, the integration of these models into clinical practice without targeted, subsite-specific validation poses unacceptable risks, including misclassification and inappropriate treatment pathways. Until rigorous, subsite-specific verification is achieved, the assumption that these models function reliably as all-in-one decision-support systems remains clinically hazardous. The analysis of clinical factors revealed that ChatGPT’s staging accuracy was significantly influenced by anatomical localization, with performance dropping markedly in complex regions like the oropharynx and paranasal sinuses. This observation is echoed by Schmidl et al. (8), who noted that the anatomical complexity of primary head and neck cases often tests the limits of AI diagnostic accuracy. Beyond these diagnostic challenges, the evolving digital oncology framework, extensively discussed in recent literature (8), suggests that AI tools are becoming essential assets not only for decision-making but also for preoperative planning and multidisciplinary tumor board (MDTB) preparation. While our study specifically evaluates staging and treatment selection, it aligns with this broader continuum where AI is expected to integrate complex data for intraoperative guidance and robotic-assisted surgical systems. However, the performance drops we observed in complex regions underscore a critical caveat: AI-driven surgical support still requires rigorous, subsite-specific human oversight to prevent the propagation of errors into automated clinical workflows.

The role of LLMs in oncology extends beyond initial diagnosis to include survivorship and patient communication. Jasmin et al. and Alabdalhussein et al. have emphasized the role of these models in improving quality of life assessments and posttreatment care (2, 22). This study complements this by focusing on the front end of the clinical pathway staging and treatment. While Diniz-Freitas et al. found that LLMs provide readable and accurate answers for oral cancer queries (16), our data suggests that for professional level clinical decision making, the models are not yet infallible. The high rate of partial recommendations in ChatGPT highlights a tendency for LLMs to omit critical details such as elective neck dissection or specific adjuvant therapy indications, a risk also identified in the systematic review by Filali Ansary and Lechien (10).

Furthermore, the comparison with other emerging models like Claude 3 (8) and DeepSeek-V3 (20, 21) suggests a rapidly shifting landscape. While this study focused on the ChatGPT-Gemini rivalry, the findings by Tellioğlu et al. suggest that the gap between these giants and newer models is narrowing (20). However, as highlighted by Hernández-Flores et al. even the most advanced models must be compared against the collective intelligence of a multidisciplinary tumor board to truly measure their clinical worth (24). The findings support the augmented intelligence model proposed by Yum et al., where LLMs serve as a checklist and secondary reviewer for the clinician rather than a primary decision maker (5).

A critical limitation identified in this study and mirrored in the work of Hajdari et al. in neuro oncology is the potential for hallucinations or the generation of confident but incorrect clinical plans (14). Although Gemini showed a statistically significant advantage in treatment planning in this study, it still produced a 0.6% false rate, which in a real world clinical setting could lead to catastrophic outcomes if not caught by a human expert. The temporal evolution of these models, as discussed by Qiu et al., suggests that accuracy will continue to improve, but the need for anatomical site specific validation remains paramount (7).

The comparative analysis demonstrates that Gemini is currently a more precise tool for treatment planning in head and neck oncology than ChatGPT, although both models are highly reliable for TNM staging. The sensitivity of these models to anatomical localization, particularly the decreased accuracy of ChatGPT in oropharyngeal and sinus malignancies, underscores the importance of subsite specific training. As we move toward a future where AI assisted decision making becomes standard (23), the results suggest that Gemini may offer a slight edge in synthesizing complex oncological data into actionable treatment protocols. Future research should focus on the longitudinal stability of these recommendations and their impact on patient outcomes when used as a real time clinical aid in the MDTB setting.

## Conclusion

This study demonstrates that LLMs, specifically ChatGPT and Gemini, have reached a high level of competency in processing complex head and neck oncology data, with both models exhibiting substantial agreement with the AJCC 8th edition staging system. However, a critical finding of this research is the statistically significant superiority of Gemini in treatment planning, suggesting that Gemini possesses a more refined ability to synthesize staging data into actionable, guideline concordant clinical recommendations. While both models were reliable for structured classification, the localization sensitivity observed particularly ChatGPT’s decreased accuracy in anatomically complex regions like the oropharynx and paranasal sinuses highlights that these tools are not yet site agnostic and require specialized validation for different head and neck subsites. Despite these promising results, several limitations must be acknowledged. First, the retrospective nature of the study and the reliance on static text based prompts may not fully capture the dynamic, multimodal nature of real world multidisciplinary tumor boards, where radiological and pathological images are integrated into decision making. Second, while Gemini showed superior treatment precision, the presence of even rare false recommendations or incomplete partial plans in both models emphasizes that AI should currently function only as a secondary clinical decision support tool, never replacing the expert judgment of an oncologist. Furthermore, a notable limitation is the rapid temporal evolution of generative AI models. While ChatGPT-4o and Gemini 1.5 Pro represented the pinnacle of LLM technology during this study’s execution, newer iterations like DeepSeek-V3 or GPT-5 may offer enhanced spatial reasoning. Nonetheless, our 180-case analysis provides a critical benchmark for how these ‘reasoning engines’ handle the high-stakes cognitive load of head and neck oncology. Future research should focus on the integration of multimodal AI models that can process imaging and histopathology slides alongside clinical text, and prospective clinical trials are needed to evaluate the impact of AI assisted decision making on actual patient outcomes and survival rates. Ultimately, as LLMs continue to evolve, they hold the potential to democratize expert level oncological knowledge, provided they are implemented within a rigorous ethical and clinical oversight framework.

In conclusion, while Gemini demonstrates a superior ability to synthesize actionable treatment protocols, both models should be viewed as auxiliary tools within a larger augmented intelligence ecosystem. Future integration of these models into MDTBs should aim for a holistic approach, linking clinical decision support with preoperative imaging and potentially intraoperative robotic systems to enhance the precision of head and neck cancer surgery.

## Data Availability

The raw data supporting the conclusions of this article will be made available by the authors, without undue reservation.

## References

[1] Lorenzi A Pugliese G Maniaci A Lechien JR Allevi F Boscolo Rizzo P . Reliability of large language models for advanced head and neck Malignancies management: a comparison between ChatGPT 4 and Gemini Advanced. Eur Arch Oto-Rhino-Laryngol. (2024) 281:5001–6. doi: 10.1007/s00405-024-08746-2. PMID: 38795148 PMC11392976

[2] Hundal J Cheema AY Mishaal M Zaheer A Jai X Maiti B . Assessing the role of large language learning models and artificial intelligence in improving oncology survivorship care: a comparative study of ChatGPT and Gemini. (2025). doi: 10.21203/rs.3.rs-6584974/v1.

[3] Kayaalp M Bölek H Yaşar HA . Confirmation of large language models in head and neck cancer staging. Diagnostics. (2025) 15:2375. doi: 10.3390/diagnostics15182375. PMID: 41008746 PMC12468830

[4] Alarifi M . Appropriateness of thyroid nodule cancer risk assessment and management recommendations provided by large language models. J Imaging Inf Med. (2025) 38(6):4324–35. doi: 10.1007/s10278-025-01454-1. PMID: 40032759 PMC12701103

[5] Yum JEI Naqvi SAA Zhou B Riaz IB . Reimagining cancer care with generative artificial intelligence: the promise of large language models. JCO Clin Cancer Inf. (2025) 9:e2500134. doi: 10.1200/cci-25-00134. PMID: 41325573

[6] Chen D Parsa R Swanson K Nunez JJ Critch A Bitterman DS . Large language models in oncology: a review. BMJ Oncol. (2025) 4:e000759. doi: 10.1136/bmjonc-2025-000759. PMID: 40519217 PMC12164365

[7] Qiu Z Jiang A Qi C Gan W Zhu L Mou W . Temporal evolution of large language models (LLMs) in oncology. J Transl Med. (2025) 23:1–17. doi: 10.1186/s12967-025-07227-2. PMID: 41188901 PMC12584546

[8] Schmidl B Hütten T Pigorsch S Stögbauer F Hoch CC Hussain T . Assessing the use of the novel tool Claude 3 in comparison to ChatGPT 4.0 as an artificial intelligence tool in the diagnosis and therapy of primary head and neck cancer cases. Eur Arch Oto-Rhino-Laryngol. (2024) 281:6099–109. doi: 10.1007/s00405-024-08828-1. PMID: 39112556 PMC11512878

[9] da Silva CCPN . Multimodal learning for lung cancer diagnosis and management: a deep learning pipeline for classification, TNM staging, and treatment protocol generation. Universidade NOVA de Lisboa (Portugal (2025). Master's thesis.

[10] Filali Ansary R Lechien JR . Clinical decision support using large language models in otolaryngology: a systematic review. Eur Arch Oto-Rhino-Laryngol. (2025) 282(8):4325–34. doi: 10.1007/s00405-025-09504-8. PMID: 40481345

[11] Ismayilov R Altundag O Gencoglu EA Aktas A Alparslan S Ozcicek A . Artificial intelligence for immunotherapy response assessment in lung cancer using PET/CT reports. Jpn J Radiol. (2025) 43:2042–50. doi: 10.1007/s11604-025-01840-3. PMID: 41091339

[12] Bresler TE Wilson T Makaryan T Pandya S Palmer K Meyer R . AI at the forefront: navigating oncologic care for six gastrointestinal cancers according to the NCCN guidelines utilizing Gemini-1.0 Ultra and ChatGPT-4. J Surg Oncol. (2025) 317–22. doi: 10.1002/jso.70005. PMID: 40536141

[13] Li H Huang J Liu K Liu J Liu Q Zhou Z . ChatGPT-4o outperforms Gemini Advanced in assisting multidisciplinary decision-making for advanced gastric cancer. Eur J Surg Oncol. (2025) 51(8):110096. doi: 10.1016/j.ejso.2025.110096. PMID: 40294561

[14] Hajdari S Farooq M Habib A Siddiqui AA Sarfraz L Habib SM . Evaluating large language models in neuro-oncology: a comparative study of accuracy, completeness, and clinical usefulness. J Clin Neurosci. (2025) 141:111597. doi: 10.2139/ssrn.5327509. PMID: 40907284

[15] Jain S Chakraborty B Agarwal A Sharma R . Performance of large language models (ChatGPT and Gemini Advanced) in gastrointestinal pathology and clinical review of applications in gastroenterology. Cureus. (2025) 17. doi: 10.7759/cureus.81618. PMID: 40322390 PMC12048130

[16] Freitas MD Pintor RM Silva ARS Warnakulasuriya S Diz Dios P . Assessing the accuracy and readability of ChatGPT-4 and Gemini in answering oral cancer queries—an exploratory study. Explor Digital Health Technol. (2024) 2:334–45. doi: 10.37349/edht.2024.00032. PMID: 41180259

[17] de Menezes Torres LM de Morais EF Fernandes Almeida DRM Pagotto LEC de Santana Santos T . The impact of the large language model ChatGPT in oral and maxillofacial surgery: a systematic review. Br J Oral Maxillofac Surg. (2025) 357–62. doi: 10.1016/j.bjoms.2025.03.006. PMID: 40251084

[18] Türe N Umurhan E Tahir E . Evaluation of research methodology generation by large language models in laryngology: a comparative analysis of ChatGPT-4.0 and Gemini 1.5 Flash. Eur Arch Oto-Rhino-Laryngol. (2025) 282(11):5739–49. doi: 10.1007/s00405-025-09656-7 40968205

[19] Marchi F Bellini E Iandelli A Sampieri C Peretti G . Exploring the landscape of AI-assisted decision-making in head and neck cancer treatment: a comparative analysis of NCCN guidelines and ChatGPT responses. Eur Arch Oto-Rhino-Laryngol. (2024) 281:2123–36. doi: 10.1007/s00405-024-08525-z. PMID: 38421392

[20] Tellioğlu B Pamuk E Külekci Ç Pamuk G Süslü N Kuşcu O . Comparative evaluation of ChatGPT-4o and DeepSeek-V3 in head and neck oncology. Acta Oto-Laryngologica. (2025) 145:1199–207. doi: 10.1080/00016489.2025.2563035 41151992

[21] Bashah A Salem A Al Waqeerah A Ghaleb E Wahan N Awad A . Evaluation of DeepSeek, Gemini, ChatGPT-4o, and Perplexity in responding to salivary gland cancer. BMC Oral Health. (2025) 25:1358. doi: 10.1186/s12903-025-06726-4. PMID: 40849657 PMC12374294

[22] Alabdalhussein A Al Khafaji MH Nadeem S Basharat M Aldallal H Mohammed MES . Large language models vs. professional resources for post-treatment quality-of-life questions in head and neck cancer: a cross-sectional comparison. Curr Oncol. (2025) 32:668. doi: 10.3390/curroncol32120668. PMID: 41440196 PMC12731422

[23] Andrew A Tizzard E . Large language models for improving cancer diagnosis and management in primary health care settings. J Medicine Surgery Public Health. (2024) 4:100157. doi: 10.1016/j.glmedi.2024.100157. PMID: 41936479

[24] Hernández Flores LA López Martínez JB Rosales de la Rosa JJ Aillaud De Uriarte D Contreras Garduño S Cortés González R . Assessment of challenging oncologic cases: a comparative analysis between ChatGPT, Gemini, and a multidisciplinary tumor board. J Surg Oncol. (2025) 1562–70. doi: 10.1002/jso.28121. PMID: 39936586

[25] Yan D Wang L Huang L Cheng K Huang Y Bao Y . Evaluating ChatGPT's recommendations for systematic treatment decisions in recurrent or metastatic head and neck squamous cell carcinoma: Perspectives from experts and junior doctors. Int J Cancer. (2025) 157:1888–97. doi: 10.1002/ijc.70001. PMID: . Epub 2025 Jul 19. 40682765 PMC12407031

[26] Vural Camalan B Doluoglu S Taraf NH Gunay MM Ozlugedik S . ChatGPT versus DeepSeek in head and neck cancer staging and treatment planning: guideline-based study. Eur Arch Otorhinolaryngol. (2025) 282:4815–24. doi: 10.1007/s00405-025-09524-4. PMID: 40523995 PMC12423241

[27] Pamuk E Bilen YE Külekci Ç Kuşcu O . ChatGPT-4 vs. multi-disciplinary tumor board decisions for the therapeutic management of primary laryngeal cancer. Acta Otolaryngol. (2025) 145:714–9. doi: 10.1080/00016489.2025.2502563. PMID: . Epub 2025 May 13. 40358250

